# Clinical practice guidelines for the foot and ankle in rheumatoid arthritis: a critical appraisal

**DOI:** 10.1186/1757-1146-6-S1-O15

**Published:** 2013-05-31

**Authors:** Kym Hennessy, James Woodburn, Martijn Steultjens

**Affiliations:** 1 Institute for Applied Health Research, School of Health & Life Sciences, Glasgow Caledonian University, UK; 2 Department of Podiatric Medicine, School of Science & Health, University of Western Sydney, Australia

## Background

Clinical practice guidelines are recommendations systematically developed to assist clinical decision-making and inform healthcare. Many rheumatoid arthritis (RA) management guidelines are available. However, these guidelines under-represent the foot and ankle, even though foot and ankle problems are common. Guidelines must be high quality to be beneficial. This study aimed to identify and critically appraise clinical practice guidelines for foot and ankle management in RA.

## Methods

Guidelines were identified electronically and through hand searching. Search terms ‘rheumatoid arthritis’, ‘clinical practice guidelines’ and related synonyms were used. Foot and ankle search terms were excluded, to ensure guidelines meeting the inclusion criteria were not precluded if foot and ankle management was not mentioned in the title or keywords. Critical appraisal and quality rating were conducted using the Appraisal of Guidelines for Research and Evaluation (AGREE) II instrument.

## Results

Twenty-two guidelines were included. Five guidelines were high quality and recommended for use. Five high quality and six low quality guidelines were recommended for use with modifications. Six low quality guidelines were not recommended for use. Two guidelines were foot and ankle specific. Five early and eleven established RA guidelines were recommended for use. Five recommendation domains were identified in early and established RA guidelines. These were multidisciplinary team care, foot healthcare access, foot health assessment/review, orthoses/insoles/splints, and therapeutic footwear. Established RA guidelines also had an ‘other treatments’ domain.

## Conclusions

Foot and ankle management for RA feature in most widely published clinical practice guidelines. Unfortunately, supporting evidence is low quality. Agreement levels are predominantly ‘expert opinion’ or ‘good clinical practice’. Clinical practice guidelines require better underpinning by high quality research evidence. Clinical relevance: Identification of recommendations from high quality guidelines for podiatric care of RA related foot and ankle issues.

